# Molecular effects of intermittent stress on primary feline uroepithelial cell culture as an *in vitro* model of feline idiopathic cystitis

**DOI:** 10.3389/fvets.2023.1258375

**Published:** 2023-11-06

**Authors:** Patrícia Hatala, Csilla Sebők, Máté Mackei, Karina Kárpáti, Péter Gálfi, Zsuzsanna Neogrády, Gábor Mátis

**Affiliations:** ^1^Division of Biochemistry, Department of Physiology and Biochemistry, University of Veterinary Medicine Budapest, Budapest, Hungary; ^2^National Laboratory of Infectious Animal Diseases, Antimicrobial Resistance, Veterinary Public Health and Food Chain Safety, University of Veterinary Medicine Budapest, Budapest, Hungary; ^3^Department of Pharmacology and Toxicology, University of Veterinary Medicine Budapest, Budapest, Hungary

**Keywords:** FIC, norepinephrine, chronic stress, uroepithelial cells, uroepithelial barrier

## Abstract

**Introduction:**

The most common cause of feline lower urinary tract disease (FLUDT) is feline idiopathic cystitis (FIC), which is a complex multifactorial disease with symptoms including stranguria, dysuria, hematuria, and pain during urination. The development of these symptoms is often triggered by stress, and in case of chronic stress, these symptoms will many times return. One of the most important stress hormones in the pathogenesis of FIC is norepinephrine (NE), as persistently elevated level of this hormone can be measured in the blood of cats with FIC. However, it is not well understood if recurrently elevated level of NE has any direct effect on urinary bladder, therefore the aim of this study was to investigate the molecular effects of intermittent NE exposure on feline primary uroepithelial cell culture.

**Methods:**

Primary uroepithelial cells were gained from the mucosa of the bladder of a euthanized cat, and were cultured for 6 days, then they were exposed to 10, 100, and 1,000 μM NE treatment for 3 × 1 h, including a 1 h long regeneration period between exposures.

**Results:**

NE was able to trigger pro-inflammatory response and oxidative stress in the uroepithelial cells by increasing the level of stromal cell derived factor 1 (SDF-1) and H_2_O_2_ in cell culture media. In addition, NE increased the permeability of the uroepithelium, since decreased glycosaminoglycan (GAG) concentration, tight junction protein claudin-4 content, and TER values were measured after the NE treatments.

**Discussion:**

Based on these results it can be concluded that recurrent stress mimicked by 3×1 h NE treatment has a direct molecular effect on the uroepithelial cells, which leads to inflammatory response, oxidative stress and decreased barrier function of the uroepithelium. Therefore, intermittent release of NE may have an important role in the pathogenesis of FIC and the results of this study may contribute to a better understanding of the development of this illness.

## 1. Introduction

Feline lower urinary tract disease (FLUTD) is one of the most common disorders in feline veterinary praxis. In case of FLUTD, the urination habits of the animal are altered, while such symptoms as hematuria, stranguria, dysuria, periuria, pain, and hypersensitivity during urination also appear. If the underlying factors are unknown, the disease is called feline idiopathic cystitis (FIC) (1). In case of the occurrence of petechial hemorrhages of bladder submucosa of symptomatic cats detectable by cystoscopy, the disease is referred as interstitial cystitis. This name also reflects the similarities in symptoms and pathogenesis to human interstitial cystitis (IC). Therfore, FIC could act as an animal model of IC (2, 3). The exact cause and pathogenesis of the disease are widely studied; however not yet fully understood. FIC is considered as a multifactorial disease, based on a complex interaction between the urinary bladder, adrenal glands, nervous- and immune system and the environment. The role of stress should be widely examined to have a more accurate understanding of the pathogenesis of the disease, since former studies strongly suggest that it may have a significant impact on the development of FIC (4–6). According to previous research the activity of tyrosine hydroxylase – which is the main regulatory enzyme of catecholamine production in the central nervous system – was increased in the norepinephrinergic nucleus, *locus coeruleus* and in the paraventricular nuclei of the hypothalamus of the cats with FIC. Consequently, increased norepinephrine (NE) level could also be measured permanently in the blood (7–9). The described increased tyrosine hydroxylase activity can be observed in chronically stress-exposed, but otherwise healthy cats as well; however, the plasma NE concentration decreased over time due to the inhibitory effect of the chronic stress-related elevated cortisol production. This inhibitory effect is missing in cats with FIC, as well as they have smaller adrenal gland, thus the cortisol production is lower than in healthy animals (7, 9–11). Prolonged, intermittent exposure to stressors results in complex changes in the brain, contributing to a stimulated emotional response. This leads to stimulation of the *locus coeruleus*, rapid and elevated sympathetic nervous system activity, and reduced action of inhibitory pathways that maintain the balance of the stress response in healthy individuals (12–16).

The main goal of the present study was to examine the molecular effects of chronic intermittent stress on inflammatory response, redox state, and barrier function of cultured feline uroepithelial cells as an *in vitro* model of the disease. The applied method for culturing primary feline uroepithelial cells was previously established by our research group, during which study the molecular effects of acute NE exposure on cultured uroepithelial cells were examined (17–19). Notwithstanding that the clinical sings of the disease can resolve within a few days without treatment, the reappearance of these symptoms is very common. Based on former studies up to 50% of cats with acute FIC will experience recurrence in 1 year, which can be related to the altered stress response of cats suffering from FIC (20). Therefore, in the present study cultured cells were exposed to intermittent, 3×1 h NE treatment, mimicking chronic recurrent stress to examine whether intermittent increased sympathetic nervous system activity leads to similar changes in uroepithelial cells as single, acute NE treatment, or the uroepithelial cells might be able to adapt to the periodically recurring elevated NE concentrations (19).

Concerning inflammatory mediators, the role of the pro-inflammatory cytokine interleukin-6 (IL-6) and the pro-inflammatory chemokine stromal cell derived factor-1 (SDF-1) was confirmed in the pathogenesis of the illness by previous studies as the levels of these mediators were elevated in the urine and the blood plasma of cats with FIC, human patients with IC, in rats with experimentally induced cystitis and also in feline urinary bladder cell culture after acute exposure to NE (19, 21–24). Since oxidative stress often develops during inflammatory processes in the urinary bladder, further increasing the production of various inflammatory mediators, it is essential to study both processes together (25–27).

The altered permeability of the bladder epithelium plays an important role in the appearance of FIC symptoms. In healthy animals the uroepithelium is covered by a glycosaminoglycan (GAG) layer, which, in addition to its barrier function, plays an important role in inhibiting the adhesion of various pathogenic bacteria; however, the uroepithelial GAG content is decreased in FIC-affected cats (28–30). In addition, electron microscopic examination of the bladder tissue revealed thinning of the urothelium and modulated expression and distribution pattern of tight junction proteins resulting in increased bladder permeability to water, urea, and various ions (2). Due to altered permeability of the bladder, hypertonic urine and its cytotoxic substances can easily reach the less resistant cell layers of the bladder, which are strongly innervated by the sensory nerves, hence inducing neurogenic inflammation, pain and the symptoms of FIC (2, 31). Therefore, investigation of the barrier function of uroepithelial cells is also essential to the more accurate understanding of the pathogenesis of FIC.

## 2. Materials and methods

### 2.1. Reagents

Unless specified otherwise, all chemicals were purchased from Merck KGaA (Darmstadt, Germany).

### 2.2. Isolation and culturing of epithelial cells from cat bladder

To target the role of intermittent stress in the pathogenesis of FIC in isolation from other factors, a feline primary bladder epithelial cell culture was prepared as an *in vitro* model of the disease. The study was approved by the Local Animal Welfare Committee of the University of Veterinary Medicine, Budapest, and it was confirmed that no ethical approval is required for the study, as the used cat was euthanized at an independent veterinary clinic and the body of the cat was donated by the owner for scientific purposes. The uroepithelial cell culture was prepared according to the protocol previously developed and described by our research group, and the cells were isolated from the bladder of a different animal in each study (17–19). In brief, the bladder, of a freshly euthanized 2 years old European short-hair male cat with no urinary tract disease, was excised and washed with Krebs-Ringer solution (Cat. No. J67591.AP, VWR, Radnor, Pennsylvania, United States) supplemented with 11.1 mM glucose, pH 7.4, followed by overnight incubation at 4°C in sterile minimal essential medium (MEM, Cat. No. M0446) containing 2.5 mg/mL dispase (Cat. No. D4693), 1% penicillin/streptomycin/fungizone and 20 mM HEPES at pH 7.4. After scraping the epithelial cells from the underlying connective tissue, they were resuspended in 0.25% (v/v) trypsin-1 mM EDTA (Cat. No. T4049) solution and incubated at 37°C for 30 min. Thereafter, cells were washed in MEM supplemented with 5% fetal bovine serum (FBS), 1% penicillin/streptomycin/fungizone, 20 mM HEPES, and finally they were resuspended in Defined Keratinocyte Medium (Cat. No. 10744019, Thermo Fisher, Waltham, Massachusetts, United States) supplemented with 0.5% gentamicin and 1% fungizone. The cell suspension was seeded on collagen-coated culture dishes at the final concentration of 10^6^ cells/ml. The cells were cultured in the presence of 5% CO_2_ at 37°C for 6 days. Culture medium was changed in every 48 h.

To evaluate pro-inflammatory mediators (IL-6 and SDF-1), oxidative stress markers (MDA, H_2_O_2_) and cell injury (LDH), cells were seeded on 24-well plates (Greiner Bio-One, Frickenhausen, Germany),with seeding volume of 0.6 mL cell suspension/well, while 96-well plates (Greiner Bio-One, Frickenhausen, Germany), seeding volume: 0.2 mL cell suspension/well, were used for monitoring the claudin-4 content, and for monitoring the viability of the cells. To investigate the transepithelial electrical resistance (TER), cells were seeded on 24-well, high-density polyester membrane inserts with 0,4 μm pore size (Greiner Bio-One, Frickenhausen, Germany), with seeding volumes of 0.2 mL in the upper chamber and 0.4 mL Defined Keratinocyte Medium in the bottom chamber.

### 2.3. Norepinephrine exposure of the cultured cells

To investigate whether the molecular changes in the urinary bladder cells that may lead to the symptoms of the disease are associated with repeatedly elevated presence of NE, chronic intermittent stress was mimicked, and on day 6 of culturing, the cells – both on 24-well and 96-well plates – were exposed to 10, 100, or 1,000 μM NE solution (Cat. No. 74480) dissolved in Defined Keratinocyte Medium at 37°C for 3×1 h (*n* = 3/group on 24-well plates and *n* = 6/group on 96-well plates). The concentrations of the applied treatment solutions were set to cover a wide range of possible NE concentrations based on our previous study. Between the pulsative NE treatments Defined Keratinocyte Medium without NE was added to the cultures for 1 h at 37°C. The last dose of NE treatment was followed by a 24 h regeneration time without NE supplementation.

After all, the supernatants of the cultures from the 24-well plates were collected, and the cells were lysed in M-PER reagent (Cat. No. 78501, Thermo Fisher, Waltham, Massachusetts, United States). Briefly, 300 μL M-PER reagent was added to each well, followed by 5 min shaking. Thereafter, the cells were scraped from the bottom of the wells and collected to Eppendorf tubes. The samples were stored at −80°C until further examination.

### 2.4. Measurements

#### 2.4.1. Investigation of the cell injury

To investigate the rate of plasma membrane damage caused by cell injury, the activity of lactate dehydrogenase (LDH) in cell culture media was measured by an enzyme kinetic photometric assay (Cat. No. 42111, Diagnosticum Ltd., Budapest, Hungary). 10 μL cell culture medium and 200 μL working reagent (containing 56 mM phosphate buffer, pH = 7.5; 1.6 mM pyruvate, and 240 μM NADH+H^+^) were mixed and then the absorbance was measured at 340 nm six times in one-minute intervals during incubation at 37°C using a Multiskan GO 3.2. reader (Thermo Fisher Scientific, Waltham, Massachusetts, United States). The enzyme activity was calculated based on the mean of the absorbance differences between consecutive time points. Further, the cell viability was monitored by CCK-8 (Cat. No. 96992) test on 96 well plate based on the manufacturer’s protocol, by adding 100 μL fresh culture medium and 10 μL CCK-8 reagent to each well. The absorbances of the resulted orange colored media were measured at 450 nm by a Multiscan GO 3.2. reader (Thermo Fisher Scientific, Waltham, Massachusetts, United States).

#### 2.4.2. Assessment of pro-inflammatory mediators IL-6 and SDF-1 with ELISA

The IL-6 and SDF-1 concentrations of cell culture media and cell lysates were assayed by feline-specific IL-6 and SDF-1 ELISA kits (Cat. No. MBS085030 and MBS049100, MyBioSource, San Diego, California, United States) according to the instructions of the manufacturer. The steps of the ELISA measurements were the same in case of both the IL-6 and SDF-1 detection (detection range IL-6: 6.25–200 pg/mL and SDF-1: 31.2–1,000 pg/mL). Briefly, 100 μL of horseradish peroxidase (HRP)-conjugate reagent was added to 50 μL of sample solutions, followed by 60 min incubation at 37°C. The wells were then washed four times, using 100 μL of washing solution per well, then 50 μL of Chromogen solution A and Chromogen solution B were added to each well and after 15 min incubation at 37°C, the reaction was stopped with 50 μL of stop solution per well. After 5 min the resultant color was read at 450 nm using a Multiskan GO 3.2 reader.

#### 2.4.3. Investigation of the redox state of the cells

As a marker of lipid peroxidation, the MDA concentration of the cell lysate was investigated with MDA Colorimetric Assay Kit (Cat. No. MAK-085). First, 300 μL fresh thiobarbituric acid stock solution was added to 100 μL cell lysate and incubated at 95°C for 1 h followed by cooling on ice for 10 min. Finally, the absorbance of the samples was read at 532 nm with a Multiskan GO 3.2 reader.

The extracellular H_2_O_2_ concentration was monitored with Amplex Red Hydrogen Peroxide Assay Kit (Cat. No. A22188, Thermo Fisher Scientific, Waltham, Massachusetts, United States). 50 μL Amplex Red working solution (composed of 100 μM Amplex Red and 0.2 U/mL HRP) was added to 50 μL cell culture media, and after 30 min incubation at room temperature the resultant fluorescence was measured with a Victor X2 2030 fluorometer (λex = 560 nm; λem = 590 nm).

#### 2.4.4. Investigation of the barrier function of uroepithelial cells

To monitor the GAG content of the cultured cells, Blyscan sulfated glycosaminoglycan assay kit (Cat. No. B1000, Biocolor Ltd., Carrickfergus, United Kingdom) was used. Briefly, 50 μL of cell culture media and 1 mL of Blyscan dye reagent (containing 1,9-dimethylmethylene blue) were mixed and then incubated for 30 min with continuous stirring. After 10 min centrifugation (1,300 × g), the supernatant was carefully removed, and the precipitate was dissolved in dissociation reagent to release the bounded dye. Finally, 200 μL from the blue colored samples was transferred to a 96-well microplate and the absorbances were read with a Multiskan GO 3.2 reader at 656 nm.

Claudin-4 content of the cells cultured on 96-well plates was measured directly in cultured cells by a feline-specific cell-based ELISA kit (Cat. No. MBS070256, MyBioSource, San Diego, California United States) following the instructions of the manufacturer. The absorbances were read at 450 nm using a Multiskan GO 3.2 reader.

To investigate the effect of NE on permeability of the cultured cell monolayer, the TERs were measured by EVOM2 epithelial Volt/Ohm meter (World Precision Instruments, Florida, United States) on 24-well plates with high-density membrane inserts (0,4 μm pore size). TERs were read directly following the final NE treatment, and also after 24 h regeneration time (culturing without NE supplementation).

### 2.5. Monitoring the morphological changes of the cell cultures after NE treatments by Giemsa staining

To monitor if there were any changes in the morphological characteristics of the cultured cells after 3 × 1 h NE treatment, Giemsa staining was used. Briefly, the cell cultures on the membrane inserts were fixed in 10% phosphate buffered saline (PBS)- formalin solution, then they were incubated in Giemsa dye solution at room temperature for 30 min, then the cells were washed with distilled water, and examined with an Olympus CXK-41 type microscope (OLYMPUS, Tokyo, Japan), equipped with a Canon Eos 1100D camera (Canon, Tokyo, Japan).

### 2.6. Statistical analysis

R v. 4.2.2 (32) program was used for statistical analysis. One-way ANOVA was performed to determine differences between means, and Dunnett’s post-hoc tests were utilized for pairwise comparisons. Before performing the statistical analysis, the data were tested for normal distribution using the Shapiro–Wilk test. Pearson’s correlation test was used to determine the correlations between different variables. Differences were considered to be significant at *p* < 0.05. All results are expressed as mean ± standard error of the mean.

## 3. Results

### 3.1. Investigation of the cell injury

**Figure 1:**
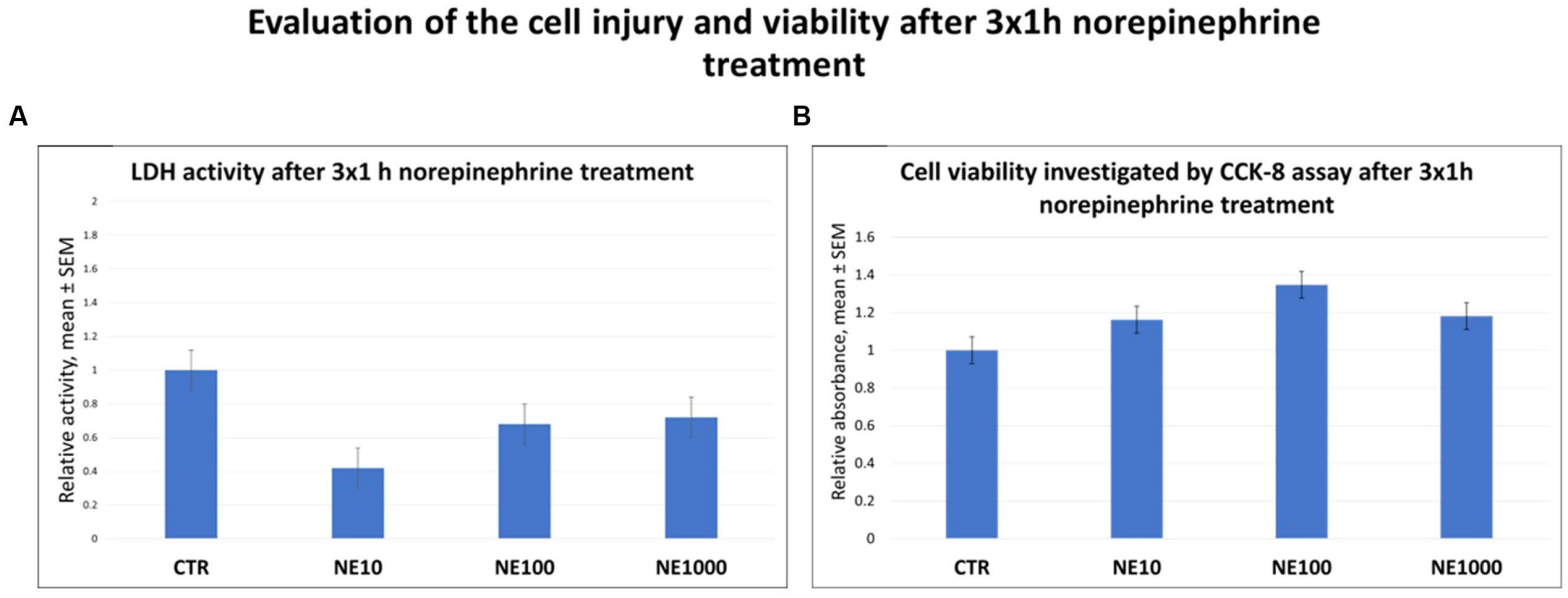
The lactate dehydrogenase (LDH) activity of the cell culture media **(A)** and investigation of cell viability by CCK-8 colorimetric assay **(B)** after 3 × 1 h norepinephrine (NE) treatment. CTR, non-treated control cells; NE10 = 10 μM norepinephrine treated cells; NE100 = 100 μM norepinephrine treated cells; NE1000 = 1,000 μM norepinephrine treated cells. *n* = 3/group, results are expressed as mean ± SEM.

No significant changes were observed in the LDH activities of the cell culture media and viability of the cells after NE treatments compared to the control groups (Figures 1A,B).

### 3.2. Assessment of pro-inflammatory mediators IL-6 and SDF-1 with ELISA

**Figure 2:**
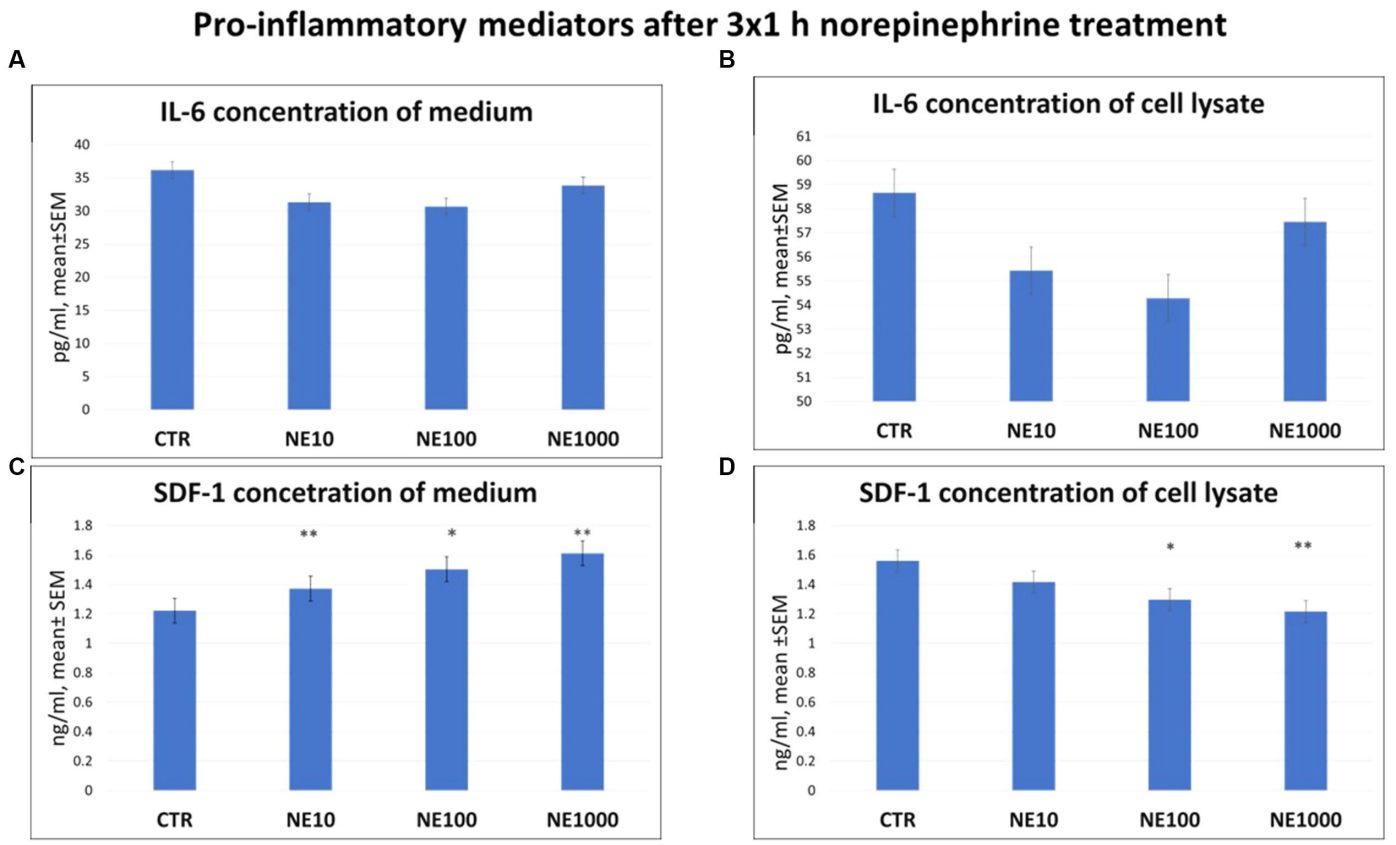
Pro-inflammatory mediators after 3×1 h norepinephrine (NE) treatment: interleukin-6 (IL-6) concentration in cell culture media **(A)**, cell lysate **(B)** and stromal cell derived factor-1 (SDF-1) concentration in cell culture media **(C)** and cell lysate **(D)**. CTR, non-treated control cells; NE10 = 10 μM norepinephrine treated cells; NE100 = 100 μM norepinephrine treated cells; NE1000 = 1,000 μM norepinephrine treated cells. *n* = 3/group, results are expressed as mean ± SEM. * *p* < 0.05, ** *p* < 0.01.

There were no significant changes in the level of IL-6, either in cell culture medium or in cell lysate after any applied NE exposure (Figures 2A,B). However, elevated SDF-1 concentrations of the media were measured in case of 3×1 h, 10, 100, and 1,000 μM NE treatment (*p* = 0.003, *p* = 0.018, *p* = 0.002, respectively, Figure 2C), and decreased SDF-1 concentrations of the cell lysates were measured after 100 and 1,000 μM NE exposure (*p* = 0.022, *p* = 0.005, respectively, Figure 2D), compared to the non-treated control wells, while no significant difference was found in case of the 10 μM NE treatment (Figure 2D).

### 3.3. Investigation of the redox state of the cells

**Figure 3:**
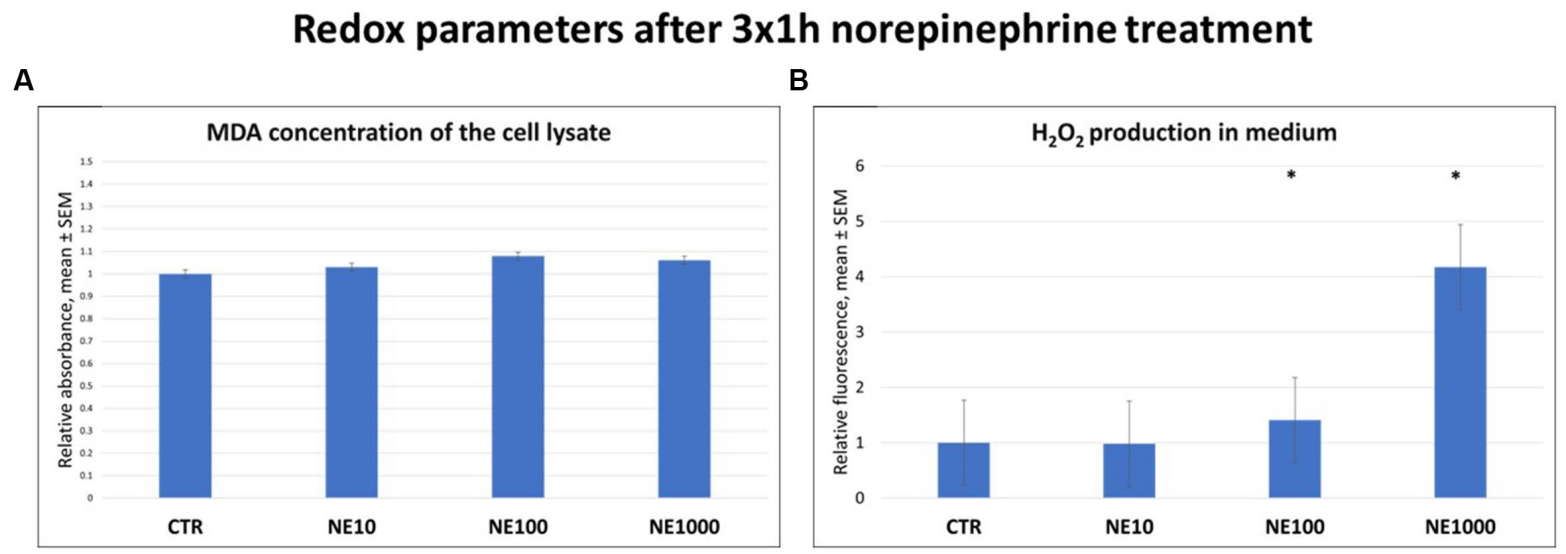
Redox parameters after 3×1 h norepinephrine (NE) treatment: malondialdehyde (MDA) **(A)** and H_2_O_2_ **(B)** concentrations. CTR, non-treated control cells; NE10 = 10 μM norepinephrine treated cells; NE100 = 100 μM norepinephrine treated cells; NE1000 = 1,000 μM norepinephrine treated cells. *n* = 3/group, results are expressed as mean ± SEM. * *p* < 0.05.

There were no significant changes in MDA levels of the cell lysates in case of any applied NE treatment (Figure 3A); however, the H_2_O_2_ production of the cultured cells, measured in the cell culture media was higher in case of 100 and 1,000 μM NE exposure compared to the non-treated controls (*p* = 0.016, *p* = 0.018, respectively, Figure 3B).

### 3.4. Investigation of the barrier function of uroepithelial cells

**Figure 4:**
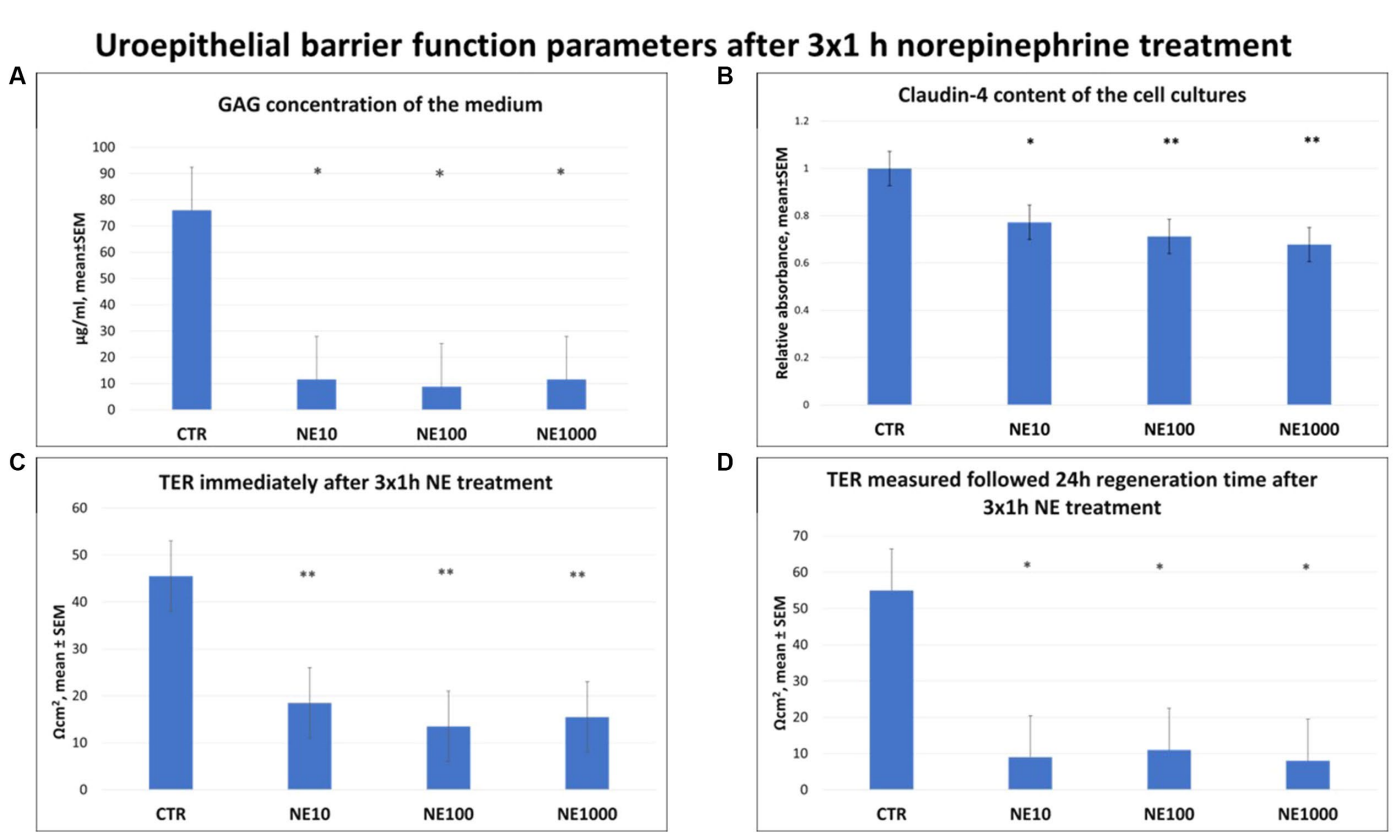
Uroepithelial barrier function parameters after 3×1 h norepinephrine (NE) treatment: glycosaminoglycan (GAG) concentration of the cell culture media **(A)**, claudin-4 content of the cells **(B)** and transepithelial electrical resistance (TER) immediately after 3×1 h NE treatment **(C)** and after 24 h regeneration time **(D)**. CTR, non-treated control cells; NE10 = 10 μM norepinephrine treated cells; NE100 = 100 μM norepinephrine treated cells; NE1000 = 1,000 μM norepinephrine treated cells. *n* = 6/group (B) and *n* = 3/group **(A,C,D)**, results are expressed as mean ± SEM. * *p* < 0.05, ** *p* < 0.01.

Significantly lower GAG concentrations were observed in the cell culture media after all the applied NE treatments (*p* = 0.024, *p* = 0.021, *p* = 0.025 for 10, 100, and 1,000 μM NE, respectively, Figure 4A). Further, significant decrease of claudin-4 content of the cultured cells was detected after each concentration of NE exposure, compared to the non-treated control cells (*p* = 0.018, *p* = 0.003, *p* = 0.001 for 10, 100, and 1,000 μM NE respectively) (Figure 4B). Similarly, TERs were also significantly lower in case of all the applied NE treatments both immediately after the treatment (*p* = 0.009, *p* = 0.009, *p* = 0.007 for 10, 100, and 1,000 μM NE, respectively, Figure 4C) and following 24 h regeneration time (*p* = 0.012, *p* = 0.016, *p* = 0.014 for 10, 100, and 1,000 μM NE, respectively) compared to the controls (Figure 4D).

### 3.5. Monitoring the morphological changes of the cell cultures after NE treatments by Giemsa staining

**Figure 5:**
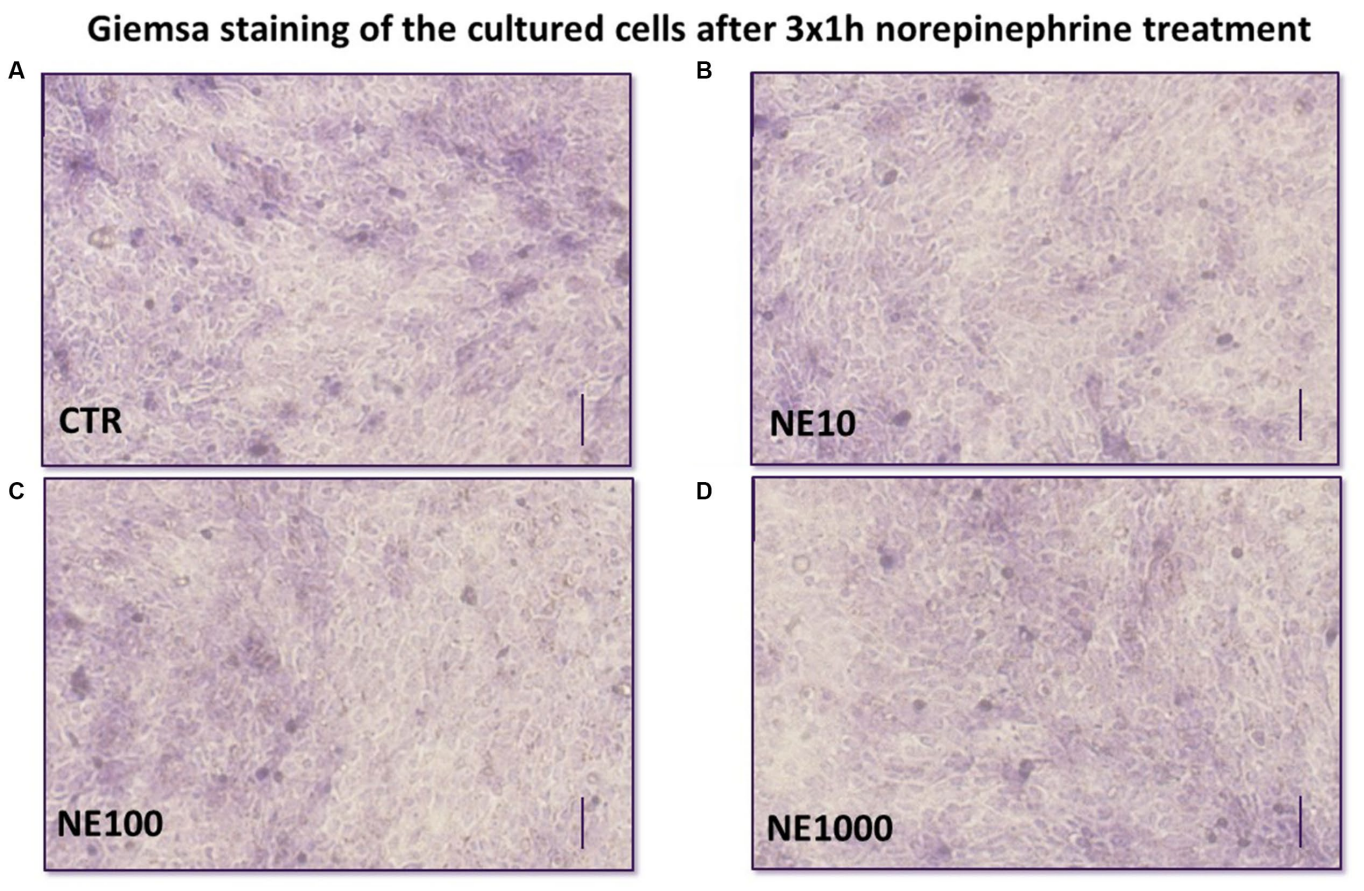
Giemsa staining of the cultured uroepithelial cells after 3×1 h norepinephrine (NE) treatment: CTR, non-treated control cells **(A)**, NE10 = 10 μM norepinephrine treated cells **(B)**, NE100 = 100 μM norepinephrine treated cells **(C)**, NE1000 = 1,000 μM norepinephrine treated cells **(D)** bar = 100 μm.

A coherent monolayer of cell cultures was formed on the membrane inserts and no morphological differences were seen in treated cells compared to control cells (Figure 5).

### 3.6. Pearson’s correlation test

A significant positive correlation was discovered via Pearson’s correlation test between SDF-1 and H_2_O_2_ content of cell culture media (*p* = 0.009, *R*^2^ = 0.51) and a significant negative correlation between SDF-1 in cell culture media and claudin-4 content of the cultured cells (*p* < 0.001, *R*^2^ = −0.63), further significant negative correlation was observed between H_2_O_2_ and claudin-4 concentrations (*p* = 0.017, *R*^2^ = −0.48) (Table 1).

**Table 1:** Correlation coefficients and *p* values as gained by Pearson’s correlation test between various parameters assessed.

Analyzed factors	Pearson correlation coefficient (*R*^2^)	*p*-value
SDF-1 medium + Claudin-4	−0.63	<0.001
SDF-1 medium + H_2_O_2_	0.51	0.009
H_2_O_2_ + Claudin-4	−0.48	0.017

## 4. Discussion

In this present study the molecular effects of intermittent NE exposure on cultured feline uroepithelial cells were investigated to examine the role of chronic, recurrent stress in the development of FIC. Since FIC is a complex multifactorial disease, it is highly beneficial to apply *in vitro* methods and to investigate the different interactions and factors (such as NE) separately or in targeted combinations to understand the development of the illness (5, 17, 18). According to former studies, NE is considered as one of the most important stress hormones involved in the pathomechanism of the disease (11). In case of increased exposure to environmental stress, the synthesis of catecholamines (such as NE) is getting stimulated together with the activation of the sympathetic nervous system (SNS). Hence, the pain fibers in the bladder wall are stimulated and Substance *P* is released, causing vasodilatation, enhanced bladder wall integrity, submucosal edema, contraction of smooth muscle, and mast cell degranulation, NE has an indirect role in the development of FIC symptoms (33). However, Birder et al. described that alfa- and beta-adrenergic receptors – as important elements of noradrenergic signaling pathways – can be found not only in the nerve cells of the bladder, but in the uroepithelial cells as well. Hence NE may possess a significant, direct effect on the stress response of the organ by stimulating the alfa-1 adrenergic receptors (34, 35). While the symptoms of acute FIC cases are usually resolved in a few days, the recurrence of them is very common, possibly in association with the altered stress response of the individual (36). Therefore, the applied NE concentrations were set to cover a wide range based on our previous studies (19), and the treatment procedure (3×1 h NE treatment followed by 24 h regeneration time) was designed to mimic a chronic intermittent SNS stress. Extracellular LDH activity was measured to assess cell damage. The method is suitable to investigate the cell injury since LDH can be found only in the cytoplasm of all cell types and it is rapidly released into the cell culture medium upon damage of the plasma membrane (37). As no significant changes were detected in present study in case of any applied NE treatment, it could be concluded that the applied NE exposures were not cytotoxic for the cultured feline uroepithelial cells. Further, the cell viability was monitored by CCK-8 colorimetric assay (38). According to the results of the present study, no significant changes observed in the metabolic activity of the cultured cells after 3×1 h NE treatment, which shows that the applied treatment procedure had no effect on cell viability.

To investigate the inflammatory role of chronic stress on the uroepithelium, the concentrations of the pro-inflammatory cytokine IL-6 and the chemokine SDF-1 were measured in cell culture media and also in cell lysates. There was no significant effect of NE treatment on the IL-6 concentration either in the cell culture media, or in the cell lysates compared to the control group. However, some previous studies have described the stimulatory effects of NE on IL-6 production in rat cardiac fibroblast cells and in human immortalized gastric epithelial cells confirming the role of NE in inflammatory processes (39, 40). Further, in the urine of women with IC, rats with experimentally induced cystitis, and cats with FIC, higher IL-6 production was detected (22, 41, 42). The concentration of SDF-1 was elevated by all applied NE treatments in the cell culture media. These results suggest that the NE-exposed cells intensely secreted this chemokine into their environment. The role of SDF-1 in the pathomechanism of the disease has been already examined, and elevated SDF-1 level was detected in the blood of cats suffering from FIC, and in the urine of human patients with IC (23, 43). Based on the present results it can be suggested that NE had a direct stimulatory effect on the pro-inflammatory processes of uroepithelial cells by increasing the level of the pro-inflammatory chemokine SDF-1, which may contribute to the development of inflammatory symptoms in cats with FIC.

Reactive oxygen and nitrogen species can be also involved in the inflammatory processes by inducing oxidative stress in cells and increasing the levels of various pro-inflammatory molecules. In addition, they also play an important role in altering cell permeability by damaging cell membranes (25–27, 44, 45). Notwithstanding that the level of MDA as an oxidative stress marker from lipid peroxidation remained unchanged after NE treatments, the concentration of H_2_O_2_ in cell culture media was increased in case of 3×1 h 100 and 1,000 μM NE exposure and the H_2_O_2_ level showed positive correlation with the extracellular SDF-1 concentration. These data are in line with the results of previous studies indicating that urinary H_2_O_2_ level was increased in patients with IC (45) and intravesical H_2_O_2_ injection was able to provoke inflammation in rat urinary bladder by increasing the amount of IL-6, IL-1 and TNF-α of the bladder wall (26). This correlation may also be caused by the oxidative stress triggered by the production of numerous reactive oxygen and nitrogen species during inflammation, and the fact that these can initiate intracellular signaling cascades contributing to increased pro-inflammatory gene expression, reflecting the strong link of the inflammatory and oxidative stress response (46–49). To evaluate the effect of intermittent stress on bladder cell integrity, GAG level of cell culture media, claudin-4 content of the cultured uroepithelial cells, and TER values were measured after NE treatment. According to numerous former studies GAG layer of the bladder wall is damaged in bladder of human patients with IC and in cats with FIC; however, it is not clear if it is a cause or consequence of FIC (1, 28, 30, 33, 50). Based on our recent results, it can be concluded that NE treatment significantly reduced the GAG content of cultured bladder epithelial cells, confirming the key role of NE in altered permeability of bladder epithelia in FIC affected cats. Furthermore, not only the GAG content, but also the abundance of claudin-4 as an important tight junction protein showed significant decrease after all the applied NE. Investigating the level of tight junction proteins is a useful tool to gain information concerning the barrier function of the uroepithelium as it is strongly maintained by the structural integrity of tight junctions. Claudin-4 is a tight junction protein, which is able to regulate the membrane permeability by forming ion channels and can be found in umbrella cells of the urinary bladder (51). Our results are in line with some earlier studies, where numerous tight junction proteins like zonula-occludens 1, occludin, E-cadherin, and uroplakins were disrupted in cats with FIC (52, 53). In addition to the above, in our research, the TER values of the uroepithelial monolayer cell cultures were significantly decreased following all used NE treatments both directly after exposure and after 24 h regeneration time, similarly to a former study, where declined TER was measured in the bladder of cats with FIC, and on cultured uroepithelial cells (2, 19). These results confirm that intermittent NE exposure can disturb the barrier function of uroepithelial cells, which may be in association with the altered GAG metabolism and diminished function of the tight junctions. In order to support that the above-mentioned results were not due to a possible morphological change caused by the NE exposure, cultures were stained with Giemsa after the treatments, and it was found that the treated cultures showed the same morphology of a confluent monolayer of uroepithelial cells as the control cell culture. In this present study negative correlations were found between claudin-4 abundance and H_2_O_2_ concentration, and between claudin-4 and SDF-1 levels, suggesting the complex interplay of the inflammatory and oxidative stress response with the impaired barrier integrity of the uroepithelium. These correlations can be explained by the results of different previous studies, where reactive oxygen species such as H_2_O_2_ was able to increase paracellular permeability by disruption of tight junction proteins. Further, the production of pro-inflammatory mediators also enhanced the uroepithelial barrier permeability (54–57).

As previously described, in cats suffering from FIC, the inhibitory effects of cortisol on catecholamine synthesis are lacking during chronic stress, therefore it is essential to examine the pathological role of NE, as a main regulatory stress hormone in the affected cats (7, 9, 11). Further, recurrence of symptoms in response to repeated stress is very common, therefore, it was investigated whether the onset of symptoms might be related to the presence of repeatedly elevated NE levels, or whether uroepithelial cells might be able to adapt to chronic stress. There are numerous studies in the literature describing the ability of certain cell types to adapt to the persistent presence of different stressors, for example hepatocytes can successfully adapt to heat stress, or endothelial cells to mechanical stress (58, 59). Although, it is described that acute and also chronic NE exposure is able to induce oxidative stress and elevated pro-inflammatory cytokine expression in rat myocardial cells (60), until now no information could be found regarding the effect of chronic presence of NE on uroepithelial cell adaptation.

Summarizing the results of the present study, it can be concluded that intermittent NE exposure mimicking chronic recurrent stress has a direct influence on cultured feline uroepithelial cells by causing pro-inflammatory cytokine release, oxidative stress and impaired uroepithelial barrier functions. Furthermore, these results are suggesting that the uroepithelial cells are not able to adapt to the intermittent elevated concentration of NE, since the chronic NE exposure resulted in similar molecular changes in the cultured cells compared to the results of a previous study of our research group, where the molecular effects of acute NE treatment were examined (19). According to these findings it can be concluded that NE might play a key role in the pathogenesis of the disease in both acute and chronic recurrent cases, and that the presence of the elevated NE concentrations in FIC affected cats may have a direct impact on the changes in the bladder that are involved in the development of disease symptoms.

## Data availability statement

The raw data supporting the conclusions of this article will be made available by the authors, without undue reservation.

## Ethics statement

The animal studies were approved by Local Animal Welfare Committee of the University of Veterinary Medicine Budapest. The studies were conducted in accordance with the local legislation and institutional requirements. Written informed consent was obtained from the owners for the participation of their animals in this study.

## Author contributions

PH: Conceptualization, Data curation, Formal analysis, Investigation, Methodology, Project administration, Visualization, Writing – original draft, Writing – review & editing. CS: Conceptualization, Data curation, Formal analysis, Investigation, Methodology, Writing – review & editing. MM: Conceptualization, Formal analysis, Funding acquisition, Investigation, Methodology, Writing – review & editing. KK: Conceptualization, Investigation, Methodology, Writing – review & editing. PG: Conceptualization, Writing – review & editing. ZN: Conceptualization, Methodology, Supervision, Writing – review & editing. GM: Conceptualization, Formal analysis, Funding acquisition, Methodology, Supervision, Writing – review & editing.
